# Development and validation of a prediction tool for intraoperative blood transfusion in brain tumor resection surgery: a retrospective analysis

**DOI:** 10.1038/s41598-023-44549-x

**Published:** 2023-10-13

**Authors:** Shugen Xiao, Fei Jiang, Yongmei Chen, Xingrui Gong

**Affiliations:** 1 Institution of Brain Disease and Neuroscience, Department of Anesthesiology, Xiangyang Central Hospital, Affiliated Hospital of Hubei University of Arts and Science, Xiangyang, Hubei China; 2 Department of Laboratory, Xiangyang Central Hospital, Affiliated Hospital of Hubei University of Arts and Science, Xiangyang, Hubei China

**Keywords:** Medical research, Risk factors

## Abstract

Early identification of a patient with a high risk of blood transfusion during brain tumor resection surgery is difficult but critical for implementing preoperative blood-saving strategies. This study aims to develop and validate a machine learning prediction tool for intraoperative blood transfusion in brain tumor resection surgery. A total of 541 patients who underwent brain tumor resection surgery in our hospital from January 2019 to December 2021 were retrospectively enrolled in this study. We incorporated demographics, preoperative comorbidities, and laboratory risk factors. Features were selected using the least absolute shrinkage and selection operator (LASSO). Eight machine learning algorithms were benchmarked to identify the best model to predict intraoperative blood transfusion. The prediction tool was established based on the best algorithm and evaluated with discriminative ability. The data were randomly split into training and test groups at a ratio of 7:3. LASSO identified seven preoperative relevant factors in the training group: hemoglobin, diameter, prothrombin time, white blood cell count (WBC), age, physical status of the American Society of Anesthesiologists (ASA) classification, and heart function. Logistic regression, linear discriminant analysis, supporter vector machine, and ranger all performed better in the eight machine learning algorithms with classification errors of 0.185, 0.193, 0.199, and 0.196, respectively. A nomogram was then established, and the model showed a better discrimination ability [0.817, 95% CI (0.739, 0.895)] than hemoglobin [0.663, 95% CI (0.557, 0.770)] alone in the test group (P = 0.000). Hemoglobin, diameter, prothrombin time, WBC, age, ASA status, and heart function are risk factors of intraoperative blood transfusion in brain tumor resection surgery. The prediction tool established using the logistic regression algorithm showed a good discriminative ability than hemoglobin alone for predicting intraoperative blood transfusion in brain tumor resection surgery.

## Introduction

Brain tumor resection with craniotomy is often a necessary step of brain tumor treatment. Due to the nature of the surgery and the highly vascularized anatomical structures of the brain and tumor, large amounts of blood loss may occur during these procedures, and this can frequently result in anemia^1,2^. Although blood transfusion can increase hemoglobin (HB) levels and improve tissue perfusion, it can result in many transfusion-related complications, such as fever, surgical site infection, acute lung injury, or even prolonged hospital stays and death^3,4^. In addition, blood shortages are an increasing problem in many countries^5^. Therefore, early identification of high-risk patients is a necessary step for implementing specific preoperative interventions to save blood resources and improve clinical outcomes.

Currently, blood transfusion guidelines suggest HB is a major indicator of blood transfusion. Blood transfusions in clinics are performed according to the HB levels^2^. However, a patient’s status, including heart function and preoperative comorbidity, affects the tolerance threshold to the blood HB level. Various prediction tools for guiding blood transfusion other than HB in other surgeries have been used in clinical settings^6,7^. A prediction tool that can quantitatively assess the probability of the necessity for intraoperative blood transfusion during brain tumor surgery has not been developed. Therefore, our study aims to develop a machine learning calculator to predict intraoperative blood transfusion in patients receiving brain tumor surgery and to evaluate its discriminative ability.

## Methods

### Ethics statement and patient selection

We performed this study following the Declaration of Helsinki, and the study was approved by the Ethics Committee of the Xiangyang Central Hospital, affiliated with the Hubei University of Arts and Science. Patient written informed consent was exempted. Patient information that could exactly identify a patient was removed prior to the data analysis.

Inclusion criteria: Patients who received elective brain tumor resection surgery in our tertiary hospital from January 2019 to December 2021 were included in this analysis. Exclusion criteria were emergency surgery; patients with hypovolemia or in shock or in a coma state; and patients who took oral anticoagulants before surgery or had participated in other clinical trials as subjects within the past 3 months. The brain tumor was diagnosed using magnetic resonance imaging (MRI). Blood transfusion was defined as receiving packed red blood cells intraoperatively. Intraoperative transfusion was conducted when the HB level was less than 70 g/L in stable patients and less than 90 g/L in patients with unstable hemodynamics. The blood transfusion decision was discussed between the anesthetist and the surgeon. If a variable had fewer than 10 events, the variable was excluded from the model establishment. Patient demographic information (gender and age), previous comorbidities (previous cerebrovascular disease, diabetes, cardiovascular disease, pulmonary disease, renal disease, and liver disease), brain tumor characteristics (diameter and number of brain tumors), patient status (American Society of Anesthesiologists (ASA) classification and heart function), and laboratory tests (white blood cell count (WBC), HB, platelet (PLT), prothrombin time (PT), activated partial prothrombin time (APTT), fibrinogen, D-dimer, total protein (TP), and albumin blood test (ALB)) were recorded. Preoperative cerebrovascular disease included ischemic and hemorrhage stroke. Previous cardiovascular disease included coronary artery stenosis > 80%, myocardial infarction, and major vascular dissection. Pulmonary disease included chronic obstructive pulmonary disease, asthma, pulmonary fibrosis, and pulmonary arterial hypertension. Renal disease included glomerulonephritis and nephrotic syndrome. Liver disease included hepatitis and cirrhosis. Tumor diameter was averaged from the length, width, and height of the tumor recorded in the electronic history database. These variables were collected for each patient from the electronic history database of our hospital and were included for the feature selection during model development and validation.

### Statistical analysis

#### Data imputation, standardization, and feature selection

**Figure 1:**
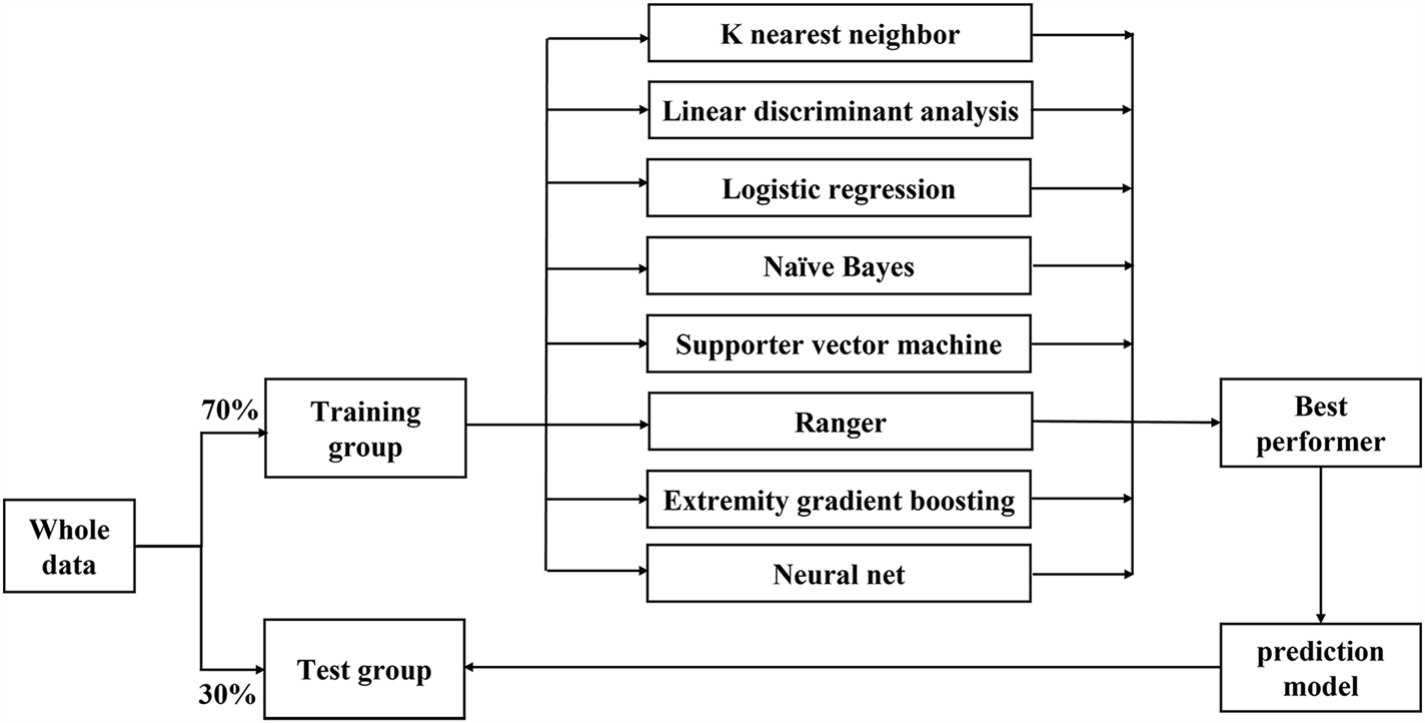
Trial flowchart.

All of the data were randomly split into training and test sets at a ratio of 7:3 (Fig. 1). The missing data were imputed using recursive partitioning and regression trees with tenfold cross-validation (CV) and then standardized to the same range of values using the max–min method in the training and test sets. Feature selection used the least absolute shrinkage and selection operator (LASSO, glmnet package in R with α = 1) with tenfold CV. LASSO shrinks the coefficients of some features, and those features with a coefficient of zero were interpreted as not being selected.

#### Machine learning algorithms and balanced performance evaluation

In our study, eight algorithms were applied to build the models and assess their predictive abilities for intraoperative blood transfusion. These algorithms included the K near neighbor algorithm (KNN)^8^, a linear discriminant analysis (LDA)^9^, a logistic regression model (LR), Naïve Bayes, support vector machine (SVM)^10^, ranger (also called randomized forest)^11^, the extremely gradient boosting machine (xgboost)^12^, and the neural net (Nnet)^13^. The machine learning performance evaluation used a tenfold CV method with 1000 iterations in the training set. The hyperparameter setting used the default hyperparameter (Supplementary Information 1). The classification error (CE) was used to evaluate the performance of the predictive ability in the training set, and the machine learning algorithm with the best performance was used for the calculator establishment. The calculator was then validated in the test set. Other measures of machine learning methods included accuracy (ACC), precision and recall area under the curve (PRAUC), area under the curve (AUC), and precision.

The quantitative data were expressed as the mean and standard division (SD) if the data were normally distributed; if not, the median and interquartile range were used. An independent t-test or Mann–Whitney *U* test was performed according to the data. The count data were expressed as their exact numbers and analyzed using the χ^2^ test. The discriminative ability between the prediction tool and HB was evaluated using the AUC of the receiver operating characteristic (ROC) curve^14^. The sample size selected for benchmarking various machine learning models met the standard of 10 events per variable^15^. The statistical analysis was performed using R software (version 4.2.2), and the machine learning models were benchmarked using the “mlr3verse” package. A *P* < 0.05 indicated statistical significance. The checklist of STROBE (Strengthening the Reporting of Observational Studies in Epidemiology) is shown in Supplementary Information 2.

### Ethics approval and consent to participate

This study was approved by the Ethics Committee of the Xiangyang Central Hospital, affiliated with the Hubei University of Arts and Science.

## Results

### Patient demographics

The patient baseline characteristics are shown in Table 1. Six patients were excluded as they took oral anticoagulants the day before surgery. Finally, a total of 541 patients who underwent brain tumor resection surgery were included in the analysis, and 141 patients (approximately 26%) received a blood transfusion. The patients in the transfused group were older than those in the non-transfused group (*P* < 0.05). Patients with an ASA physical status III or heart function II had a higher incidence of transfusion than those with an ASA physical status I or II or heart function I (*P* < 0.05). The tumor diameter, PT, and WBC were higher in the transfused group than in the non-transfused group (*P* < 0.05). Patient demographic information (gender and weight), previous co-morbidities (previous cerebrovascular disease, diabetes, cardiovascular disease, pulmonary disease, renal disease, and/or liver disease), tumor characteristics (multi-site brain tumor), and laboratory tests (PLT, activated APTT, fibrinogen, D-dimer, TP, and ALB were not different between the transfused and non-transfused groups (*P* > 0.05).

**Table 1:** Patients baseline characteristics.

	Total	Non-transfused	Transfused	P
Gender				1.000
Male	325 (60.1%)	240 (73.8%)	85 (26.2%)	
Female	216 (39.9%)	160 (74.1%)	56 (25.9%)	
Age, mean (SD)	54.5 (± 12.3)	53.5 (± 12.3)	57.5 (± 12.1)	0.002
Weight, mean (SD)	63.1 (± 13.6)	64.1 (± 12.4)	60.4 (± 16.2)	0.059
Multi-site tumor				0.680
One-site	508 (93.9%)	374 (73.6%)	134 (26.4%)	
Multi-site	33 (6.1%)	26 (78.8%)	7 (21.2%)	
Diameter, mean (SD)	3.1 (± 1.2)	2.9 (± 1.1)	3.7 (± 1.3)	< 0.001
Cerebrovascular disease				0.181
None	351 (64.9%)	266 (75.8%)	85 (24.2%)	
Present	190 (35.1%)	134 (70.5%)	56 (29.5%)	
Cardiovascular disease				1.000
None	530 (98.0%)	392 (74.0%)	138 (26.0%)	
Present	11 (2.0%)	8 (72.7%)	3 (27.3%)	
Pulmonary disease				0.211
None	510 (94.3%)	380 (74.5%)	130 (25.5%)	
Present	31 (5.7%)	20 (64.5%)	11 (35.5%)	
Hepatic disease				0.660
None	512 (94.6%)	377 (73.6%)	135 (26.4%)	
Present	29 (5.4%)	23 (79.3%)	6 (20.7%)	
Same site surgery history				0.095
None	509 (94.1%)	372 (73.1%)	137 (26.9%)	
Present	32 (5.9%)	28 (87.5%)	4 (12.5%)	
DM				0.420
None	507 (93.7%)	377 (74.4%)	130 (25.6%)	
Present	34 (6.3%)	23 (67.6%)	11 (32.4%)	
ASA				< 0.001
I ~ II	372 (68.8%)	292 (78.5%)	80 (21.5%)	
III	169 (31.2%)	108 (63.9%)	61 (36.1%)	
Heart function				0.030
I	388 (71.7%)	297 (76.5%)	91 (23.5%)	
II	153 (28.3%)	103 (67.3%)	50 (32.7%)	
WBC, mean (SD)	6.1 (± 2.3)	5.9 (± 2.2)	6.7 (± 2.3)	< 0.001
HB, mean (SD)	130.4 (± 16.4)	133.8 (± 14.0)	120.9 (± 18.9)	< 0.001
PLT, mean (SD)	216.9 (± 65.6)	216.2 (± 61.9)	218.9 (± 75.3)	0.570
PT, mean (SD)	13.1 (± 0.8)	13.0 (± 0.7)	13.2 (± 0.9)	0.017
APTT, mean (SD)	35.9 (± 3.8)	36.1 (± 3.9)	35.6 (± 3.7)	0.181
Fibrinogen, mean (SD)	3.0 (± 0.8)	3.0 (± 0.8)	3.0 (± 0.8)	0.891
D-dimer, mean (SD)	0.5 (± 1.6)	0.5 (± 1.7)	0.6 (± 1.1)	0.121
TP, mean (SD)	66.6 (± 5.7)	66.7 (± 5.4)	66.4 (± 6.4)	0.710
ALB, mean (SD)	42.1 (± 3.9)	42.2 (± 3.8)	41.7 (± 4.1)	0.392
NT-proBNP, mean (SD)	50.4 (± 101.7)	48.0 (± 76.6)	57.4 (± 153.6)	0.471

Results are expressed as mean (SD) for continuous data and n (proportion) for categorical data. *APTT* Activated partial thromboplastin time, *ASA* American Society of Anesthesiologists, *DM* Diabetes Mellitus, *HB* Hemoglobin, *PLT* Platelet, *PT* Prothrombin time, *TP* Total protein, *ALB* Albumin, *WBC* White blood cell.

### Feature selection and machine learning model performance evaluation

**Figure 2:**
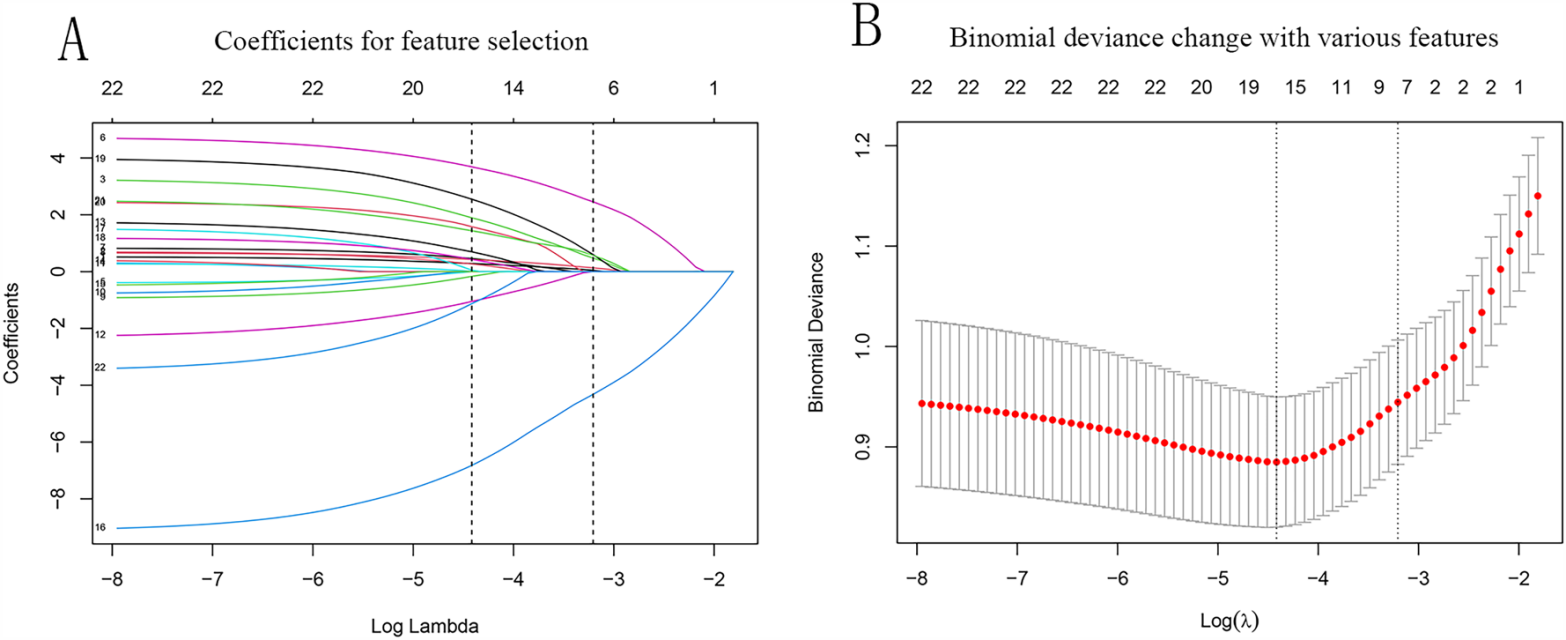
Results of the least absolute shrinkage and selection operator analysis for the feature selection on the training group. (**A**) With a decreasing log(λ), the influence of a variable that enters the model earlier is greater than that enters later. Each color line represents one of the explanatory variables. The plots demonstrate the extent that a variable enters the model and influences the response. (**B**) Binomial deviance changes with the values of λ are shown, and the acceptable variables are indicated by λ min to 1se. Seven features, namely hemoglobin, diameter, prothrombin time, WBC, age, the physical status of the American Society of Anesthesiologists classification, and heart function, were identified before the λ1se. Lines 1–22 indicate the exploratory variables: [1] ASA, [2] DM, [3] age, [4] cardiovascular disease, [5] cerebrovascular disease, [6] diameter, [7] gender, [8] heart function, [9] hepatic disease, [10] multi-site tumor, [11] pulmonary disease, [12] surgery history of same site, [13] ALB, [14] APTT, [15] D-dimer, [16] HB, [17] NT-proBNP, [18] PLT, [19] PT, [20] TP, [21] WBC, and [22] fibrinogen.

**Figure 3:**
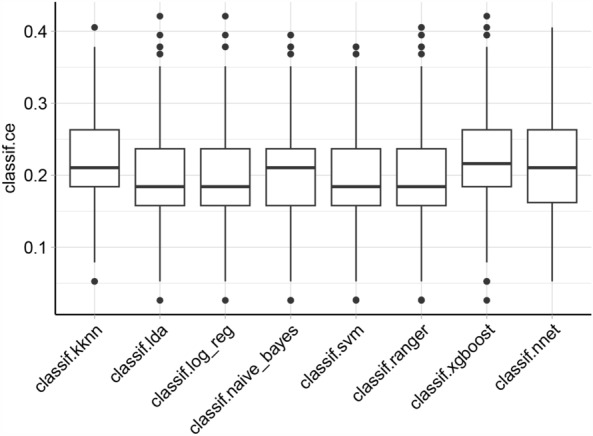
Boxplot of the classification errors of the eight machine learning algorithms. *KNN* K near neighbor, *LDA* linear discriminant analysis, *LR* logistic regression, Naïve Bayes, *SVM* supporter vector machine, ranger, *xgboost* extremely gradient boosting model, *Nnet* neural net.

LASSO identified seven variables, and the relevant plots are shown in Fig. 2A and B. In our case, λ1se indicated that these variables were related to intraoperative blood transfusion and were chosen for model establishment: HB, diameter, PT, WBC, age, the physical status of the ASA classification, and heart function. HB and WBC had three missing values, and among the seven variables of interest, they were imputed using recursive partitioning and the regression trees algorithm prior to machine learning model benchmarking. The variance inflation factors for HB, diameter, PT, WBC, age, ASA, and heart function were 1.20, 1.18, 1.05, 1.08, 1.22, 1.20, and 1.19, respectively. The performance evaluation results of the various machine learning models showed that the CE values using KNN, LDA, LR, Naïve Bayes, SVM, ranger, xgboost, and Nnet were 0.217, 0.193, 0.185, 0.204, 0.199, 0.196, 0.244, and 0.199, respectively. The boxplot is shown in Fig. 3. Values of the CE, AUC, ACC, PRAUC, and precision for each machine learning method are shown in Table 2. The results showed that the LR, LDA, SVM, and ranger models performed better than the other four algorithms (Wilcox test, *P* = 0.000).

**Table 2:** Performance evaluation of eight machine learning algorithms.

	CE	AUC	ACC	PRAUC	Precision
KNN	0.217	0.776	0.783	0.585	0.643
LDA	0.193	0.826	0.807	0.678	0.706
LR	0.185	0.828	0.815	0.682	0.736
Naïve Bayes	0.204	0.815	0.796	0.617	0.653
SVM	0.199	0.799	0.801	0.646	0.721
Ranger	0.196	0.825	0.804	0.689	0.732
Xgboost	0.244	0.755	0.756	0.596	0.547
Nnet	0.199	0.775	0.802	0.610	0.728

*ACC* Accuracy, *AUC* Area under the curve, *CE* Classification error, *Xgboost* Extremely gradient boosting machine, *KNN* K near neighbor algorithm, *LDA* Linear discriminant analysis, *LR* Logistic regression, *PRAUC* Precision and recall area under the curve, *Nnet* Neural net, *SVM* Support vector machine.

### Prediction tool establishment and performance evaluation

**Figure 4:**
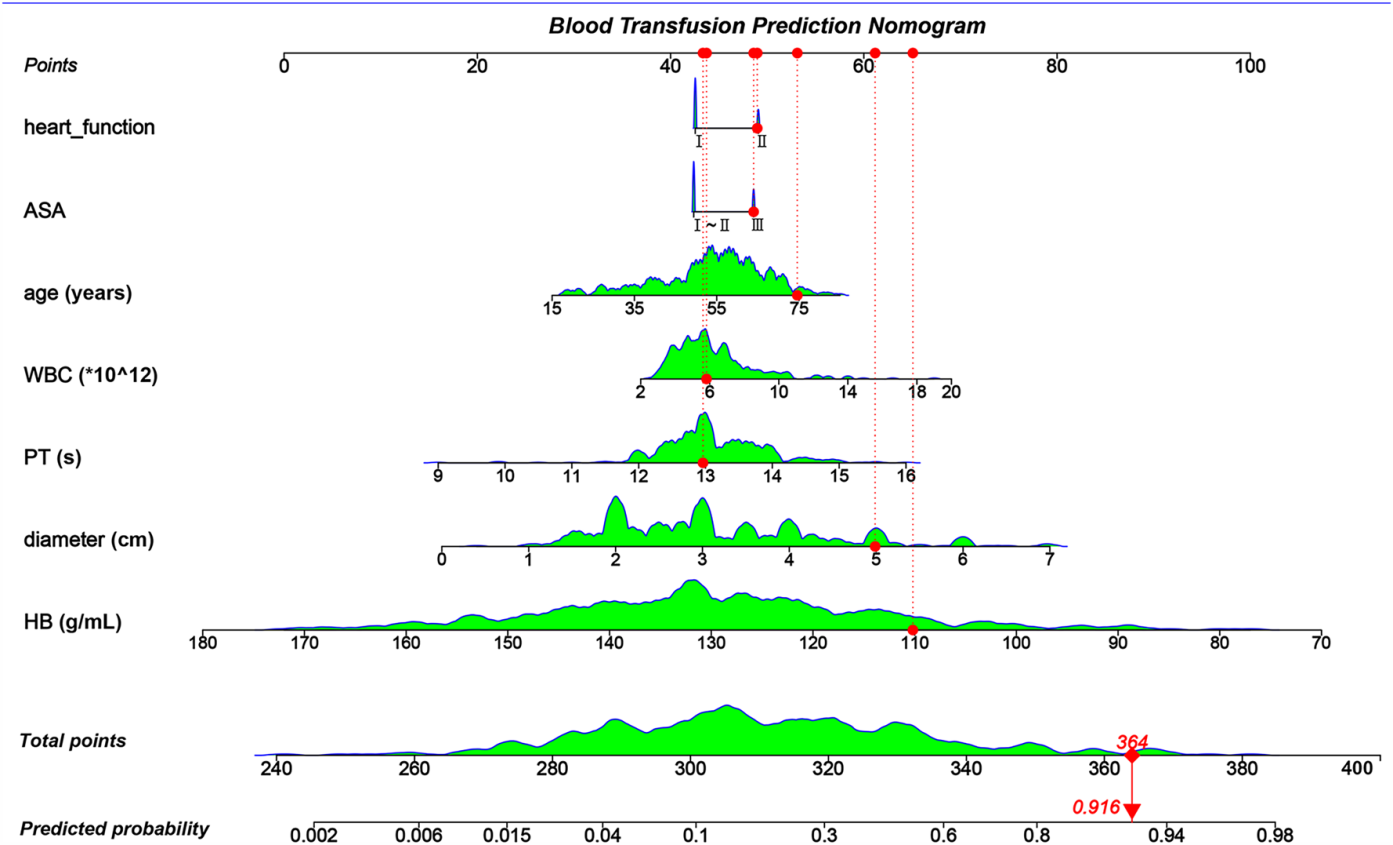
Prediction tool to predict the probability of intraoperative blood transfusion in a patient who underwent brain tumor surgery. For example, a 75-year-old patient who underwent a 5 cm brain tumor resection surgery with ASA III, heart function II, WBC (6 × 10^12^), PT (13S), and HB (110 g/L) had a total score of 364 in terms of these seven variables, and the probability of intraoperative blood transfusion was 91.6%. *ASA* American Society of Anesthesiologists, *WBC* white blood cell, *HB* hemoglobin, *PLT* Platelet.

Considering the advantages of LR in terms of ease of implementation and understanding, as well as its comparable predictive ability to machine learning algorithms, LR was chosen to establish the nomogram. The prediction tool was established with the seven identified independent relative factors, namely HB, diameter, PT, WBC, age, ASA, and heart function (Fig. 4). Briefly, to understand the prediction tool, we first acquired the patient’s baseline values of the seven variables. We then obtained the points of each variable from the first line and added all the scores. The total score predicted the probability of blood transfusion in patients receiving brain tumor surgery. The nomogram was then validated in the test set and the CE was 19.0%, similar with that of CE in the train set. An online calculator has been created (https://gongxrhbxysimple.shinyapps.io/dynnomapp/. Values of 1 and 2 represent ASA I–II and III, respectively).

**Figure 5:**
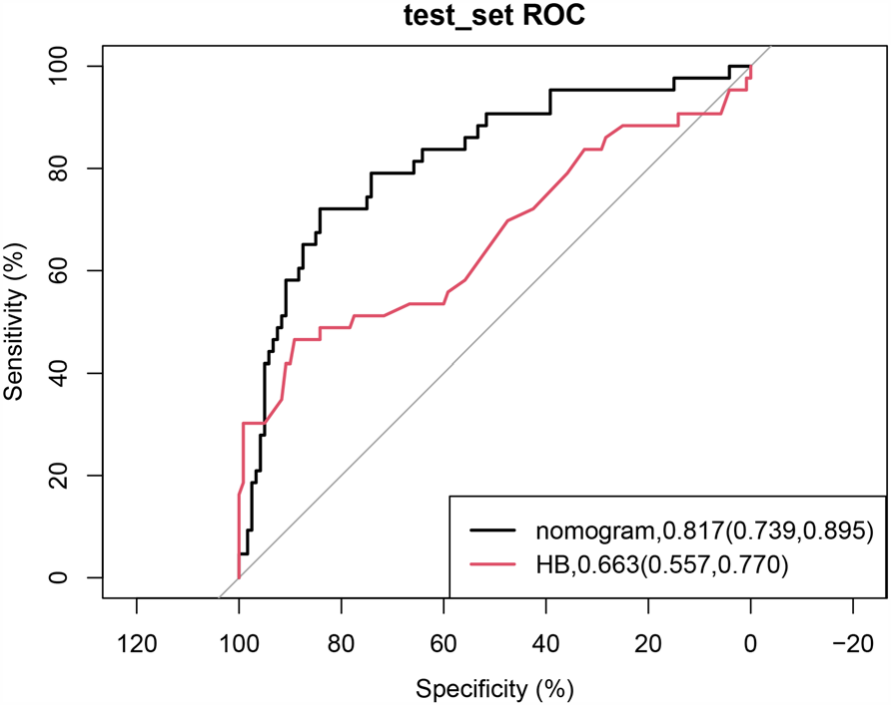
ROC curve of the prediction tool and HB of the probability of intraoperative blood transfusion in patients who underwent brain tumor surgery in the test group.

In addition, we then compared the nomogram with HB for predicting the blood transfusion. The confusion matrices showed that the nomogram was superior to HB for predicting the blood transfusion in the test set (Table 3; χ^2^ = 36.76, P = 1.339e−09, Yates' continuity correction). The AUC value of the predicted probability using a prediction tool [0.817, 95% CI (0.739, 0.895)] was better than that of HB [0.663, 95% CI (0.557, 0.770)] in the test group (P = 0.000, Fig. 5). In addition, the Hosmer–Lemeshow test of concordance between the virtual and predicted probability with prediction tool (χ^2^ = 10.97, P = 0.278) was better than that of HB (χ^2^ = 27.94, P = 0.001) alone.

**Table 3:** Confusion matrix for comparison the predictive ability of nomogram and HB.

	HB	
	No-transfused	Transfused
Nomogram		
No-transfused	137	2
Transfused	15	9

Pearson's Chi-squared test with Yates' continuity correction: χ^2^ = 36.76, df = 1, P = 1.339e−09

*HB* Hemoglobin.

## Discussion

In this study, approximately one-fourth of the patients who underwent craniotomy for brain tumor removal received an intraoperative blood transfusion. Seven preoperative indicators, namely HB, diameter, PT, WBC, age, ASA classification, and heart function, were identified as relevant factors for intraoperative blood transfusion during brain tumor resection surgery. We established a calculator to predict intraoperative blood transfusion, and the results showed that the prediction model had a good discriminative ability.

Feature selection removes the redundant features, and this makes the model much easier to interpret and generalize. The selection process of the shrinking ability of this operator depends on modifying the absolute value of the coefficient of functions. In this study, we used the LASSO classifier and identified seven relative factors for intraoperative blood transfusion. In addition, machine learning algorithms have been increasingly used in the anesthesiology^16,17^ and pain^18^ fields. We included eight machine learning methods and acquired a balanced performance evaluation by benchmarking various algorithms. Machine learning algorithms establish complex models and make accurate decisions when given relevant data. Our results suggest that conventional LR-based analyses can perform comparably to machine learning techniques. This might be attributable to the structure and dimensions of our data.

Given its strong interpretability and practical application in a clinical setting, we chose LR as the method to establish our prediction tool. The discriminative ability was assessed using the AUC in the test set. The AUC was over 0.8 and better than that of HB alone, indicating the good performance of the prediction tool. By calculating the points of the seven preoperative variables with the prediction tool, surgeons can easily assess the risk of intraoperative blood transfusion prior to surgery. Thus, preoperative blood-saving strategies can be considered to reduce the risk of blood transfusion for high-risk patients and to decrease the economic burden for low-risk families.

Tumor diameter was identified as an independent risk factor for intraoperative blood transfusion in our study. A big tumor typically has a fruitful blood supply and makes the surgery more difficult. High-grade tumors are more aggressive, large, less differentiated, and therefore more difficult to extract. The PT reflects the plasma levels of fibrinogen along with the coagulation factors II, V, VII, and X. Our results suggested that PT is an important indicator of intraoperative blood transfusion. The reason may be because prolonged PT decreases blood clots and results in massive blood loss^19–21^. Hemostasis disturbances during craniotomies are multifactorial and frequently comorbid with hyperfibrinolysis, abundant tissue factor expression, and the loss of coagulation factors^22^. Thus, our study identified an abnormal PT as a risk factor for intraoperative blood transfusion.

Our results also showed that age, WBC, ASA, and heart function are risk factors for intraoperative blood transfusion. Elderly patients are likely to have decreased hematopoietic activity, decreased platelet function, and a lower transfusion threshold and are frequently comorbid with cardiovascular and cerebrovascular diseases, which make critical organ tissue sensitive to ischemia. However, maintaining a higher HB level is an effective method to provide sufficient oxygen delivery during major surgery^23–25^. WBC indicates an inflammatory response in the body and may disrupt the coagulation process and result in increased blood loss during a surgical procedure^26^. The ASA physical status and heart function reflect the patient’s physical status, comorbidity, and activity tolerance before surgery, and patients in poorer conditions require higher blood HB levels to maintain hemodynamic stability^27^.

We established this prediction tool for predicting intraoperative blood transfusion using seven variables, and the prediction tool is simple and easy to use. However, the present study has some shortcomings: (1) This was a retrospective study, and some of the variables that may have affected the transfusion could not be measured. (2) The prediction tool was established based on our single center, and the prediction ability of this model needs to be confirmed by external tests in the future. However, the common preoperative variables make the prediction tool easy to use in clinics.

## Conclusions

This study identified seven risk factors, namely preoperative HB, tumor diameter, PT, WBC, age, the physical status of the ASA classification, and heart function, that affect intraoperative blood transfusion during brain tumor resection surgery. Based on the seven indicators, a prediction tool was established, and this tool enables an accurate assessment of the probability of intraoperative blood transfusion during brain tumor resection surgery (Supplementary Information).

### Supplementary Information

Supplementary Information 1.

Supplementary Information 2.

## Data Availability

The datasets analyzed during the current study are available from the corresponding author upon reasonable request.

## Abbreviations

ALB: Albumin
APTT: Activated partial prothrombin time
ASA: American Society of Anesthesiologists
AUC: Area under the curve
HB: Hemoglobin
LASSO: Least absolute shrinkage and selection operator
MRI: Magnetic resonance imaging
PLT: Platelet
PRAUC: Precision and recall area under the curve
PT: Prothrombin time
ROC: Receiver operating characteristic
TP: Total protein
WBC: White blood cell
