# Supplementary material for: Development and validation of a prediction tool for intraoperative blood transfusion in brain tumor resection surgery: a retrospective analysis
Source: Sci Rep. 2023 Oct 13;13:17428. doi: 10.1038/s41598-023-44549-x (PMC10575918; doi:10.1038/s41598-023-44549-x)
Supplement: Supplementary file 1 — Supplementary Information 1. [file 41598_2023_44549_MOESM1_ESM.docx]

Parameters of the machine learning algorithms were exported from “mlr3” package and were listed below:

<param_set>"classif.kknn"

id class lower upper nlevels default value

1: k ParamInt 1 Inf Inf 7 7

2: distance ParamDbl 0 Inf Inf 2

3: kernel ParamFct NA NA 10 optimal

4: scale ParamLgl NA NA 2 TRUE

5: ykernel ParamUty NA NA Inf

6: store_model ParamLgl NA NA 2 FALSE

<ParamSet>"classif.lda"

id class lower upper nlevels default parents value

1: dimen ParamUty NA NA Inf <NoDefault[3]>

2: method ParamFct NA NA 4 moment

3: nu ParamInt -Inf Inf Inf <NoDefault[3]> method

4: predict.method ParamFct NA NA 3 plug-in

5: predict.prior ParamUty NA NA Inf <NoDefault[3]>

6: prior ParamUty NA NA Inf <NoDefault[3]>

7: tol ParamDbl -Inf Inf Inf <NoDefault[3]>

<ParamSet>"classif.log_reg"

id class lower upper nlevels default value

1: dispersion ParamUty NA NA Inf

2: epsilon ParamDbl -Inf Inf Inf 1e-08

3: etastart ParamUty NA NA Inf <NoDefault[3]>

4: maxit ParamDbl -Inf Inf Inf 25

5: model ParamLgl NA NA 2 TRUE

6: mustart ParamUty NA NA Inf <NoDefault[3]>

7: offset ParamUty NA NA Inf <NoDefault[3]>

8: singular.ok ParamLgl NA NA 2 TRUE

9: start ParamUty NA NA Inf

10: trace ParamLgl NA NA 2 FALSE

11: x ParamLgl NA NA 2 FALSE

12: y ParamLgl NA NA 2 TRUE

<ParamSet>"classif.naive_bayes"

id class lower upper nlevels default value

1: eps ParamDbl -Inf Inf Inf 0

2: laplace ParamDbl 0 Inf Inf 0

3: threshold ParamDbl -Inf Inf Inf 0.001

<ParamSet>"classif.svm"

id class lower upper nlevels default parents value

1: cachesize ParamDbl -Inf Inf Inf 40

2: class.weights ParamUty NA NA Inf

3: coef0 ParamDbl -Inf Inf Inf 0 kernel

4: cost ParamDbl 0 Inf Inf 1 type

5: cross ParamInt 0 Inf Inf 0

6: decision.values ParamLgl NA NA 2 FALSE

7: degree ParamInt 1 Inf Inf 3 kernel

8: epsilon ParamDbl 0 Inf Inf 0.1

9: fitted ParamLgl NA NA 2 TRUE

10: gamma ParamDbl 0 Inf Inf <NoDefault[3]> kernel

11: kernel ParamFct NA NA 4 radial

12: nu ParamDbl -Inf Inf Inf 0.5 type

13: scale ParamUty NA NA Inf TRUE

14: shrinking ParamLgl NA NA 2 TRUE

15: tolerance ParamDbl 0 Inf Inf 0.001

16: type ParamFct NA NA 2 C-classification

<ParamSet>"classif.ranger"

id class lower upper nlevels default parents value

1: alpha ParamDbl -Inf Inf Inf 0.5

2: always.split.variables ParamUty NA NA Inf <NoDefault[3]>

3: class.weights ParamUty NA NA Inf

4: holdout ParamLgl NA NA 2 FALSE

5: importance ParamFct NA NA 4 <NoDefault[3]>

6: keep.inbag ParamLgl NA NA 2 FALSE

7: max.depth ParamInt 0 Inf Inf

8: min.node.size ParamInt 1 Inf Inf

9: min.prop ParamDbl -Inf Inf Inf 0.1

10: minprop ParamDbl -Inf Inf Inf 0.1

11: mtry ParamInt 1 Inf Inf <NoDefault[3]>

12: mtry.ratio ParamDbl 0 1 Inf <NoDefault[3]>

13: num.random.splits ParamInt 1 Inf Inf 1 splitrule

14: num.threads ParamInt 1 Inf Inf 1 1

15: num.trees ParamInt 1 Inf Inf 500

16: oob.error ParamLgl NA NA 2 TRUE

17: regularization.factor ParamUty NA NA Inf 1

18: regularization.usedepth ParamLgl NA NA 2 FALSE

19: replace ParamLgl NA NA 2 TRUE

20: respect.unordered.factors ParamFct NA NA 3 ignore

21: sample.fraction ParamDbl 0 1 Inf <NoDefault[3]>

22: save.memory ParamLgl NA NA 2 FALSE

23: scale.permutation.importance ParamLgl NA NA 2 FALSE importance

24: se.method ParamFct NA NA 2 infjack

25: seed ParamInt -Inf Inf Inf

26: split.select.weights ParamUty NA NA Inf

27: splitrule ParamFct NA NA 3 gini

28: verbose ParamLgl NA NA 2 TRUE

29: write.forest ParamLgl NA NA 2 TRUE

id class lower upper nlevels default parents value

<ParamSet>"classif.xgboost"

id class lower upper nlevels default parents value

1: alpha ParamDbl 0 Inf Inf 0

2: approxcontrib ParamLgl NA NA 2 FALSE

3: base_score ParamDbl -Inf Inf Inf 0.5

4: booster ParamFct NA NA 3 gbtree

5: callbacks ParamUty NA NA Inf <list[0]>

6: colsample_bylevel ParamDbl 0 1 Inf 1

7: colsample_bynode ParamDbl 0 1 Inf 1

8: colsample_bytree ParamDbl 0 1 Inf 1

9: disable_default_eval_metric ParamLgl NA NA 2 FALSE

10: early_stopping_rounds ParamInt 1 Inf Inf

11: early_stopping_set ParamFct NA NA 3 none none

12: eta ParamDbl 0 1 Inf 0.3

13: eval_metric ParamUty NA NA Inf <NoDefault[3]>

14: feature_selector ParamFct NA NA 5 cyclic booster

15: feval ParamUty NA NA Inf

16: gamma ParamDbl 0 Inf Inf 0

17: grow_policy ParamFct NA NA 2 depthwise tree_method

18: interaction_constraints ParamUty NA NA Inf <NoDefault[3]>

19: iterationrange ParamUty NA NA Inf <NoDefault[3]>

20: lambda ParamDbl 0 Inf Inf 1

21: lambda_bias ParamDbl 0 Inf Inf 0 booster

22: max_bin ParamInt 2 Inf Inf 256 tree_method

23: max_delta_step ParamDbl 0 Inf Inf 0

24: max_depth ParamInt 0 Inf Inf 6

25: max_leaves ParamInt 0 Inf Inf 0 grow_policy

26: maximize ParamLgl NA NA 2

27: min_child_weight ParamDbl 0 Inf Inf 1

28: missing ParamDbl -Inf Inf Inf NA

29: monotone_constraints ParamUty NA NA Inf 0

30: normalize_type ParamFct NA NA 2 tree booster

31: nrounds ParamInt 1 Inf Inf <NoDefault[3]> 1

32: nthread ParamInt 1 Inf Inf 1 1

33: ntreelimit ParamInt 1 Inf Inf

34: num_parallel_tree ParamInt 1 Inf Inf 1

35: objective ParamUty NA NA Inf binary:logistic

36: one_drop ParamLgl NA NA 2 FALSE booster

37: outputmargin ParamLgl NA NA 2 FALSE

38: predcontrib ParamLgl NA NA 2 FALSE

39: predictor ParamFct NA NA 2 cpu_predictor

40: predinteraction ParamLgl NA NA 2 FALSE

41: predleaf ParamLgl NA NA 2 FALSE

42: print_every_n ParamInt 1 Inf Inf 1 verbose

43: process_type ParamFct NA NA 2 default

44: rate_drop ParamDbl 0 1 Inf 0 booster

45: refresh_leaf ParamLgl NA NA 2 TRUE

46: reshape ParamLgl NA NA 2 FALSE

47: sample_type ParamFct NA NA 2 uniform booster

48: sampling_method ParamFct NA NA 2 uniform booster

49: save_name ParamUty NA NA Inf

50: save_period ParamInt 0 Inf Inf

51: scale_pos_weight ParamDbl -Inf Inf Inf 1

52: seed_per_iteration ParamLgl NA NA 2 FALSE

53: skip_drop ParamDbl 0 1 Inf 0 booster

54: strict_shape ParamLgl NA NA 2 FALSE

55: subsample ParamDbl 0 1 Inf 1

56: top_k ParamInt 0 Inf Inf 0 booster,feature_selector

57: training ParamLgl NA NA 2 FALSE

58: tree_method ParamFct NA NA 5 auto booster

59: tweedie_variance_power ParamDbl 1 2 Inf 1.5 objective

60: updater ParamUty NA NA Inf <NoDefault[3]>

61: verbose ParamInt 0 2 3 1 0

62: watchlist ParamUty NA NA Inf

63: xgb_model ParamUty NA NA Inf

id class lower upper nlevels default parents value

<ParamSet>"classif.nnet"

id class lower upper nlevels default value

1: Hess ParamLgl NA NA 2 FALSE

2: MaxNWts ParamInt 1 Inf Inf 1000

3: Wts ParamUty NA NA Inf <NoDefault[3]>

4: abstol ParamDbl -Inf Inf Inf 1e-04

5: censored ParamLgl NA NA 2 FALSE

6: contrasts ParamUty NA NA Inf

7: decay ParamDbl -Inf Inf Inf 0

8: mask ParamUty NA NA Inf <NoDefault[3]>

9: maxit ParamInt 1 Inf Inf 100

10: na.action ParamUty NA NA Inf <NoDefault[3]>

11: rang ParamDbl -Inf Inf Inf 0.7

12: reltol ParamDbl -Inf Inf Inf 1e-08

13: size ParamInt 0 Inf Inf 3 3

14: skip ParamLgl NA NA 2 FALSE

15: subset ParamUty NA NA Inf <NoDefault[3]>

16: trace ParamLgl NA NA 2 TRUE
