# Supplementary material for: Development and validation of a prediction tool for intraoperative blood transfusion in brain tumor resection surgery: a retrospective analysis
Source: Sci Rep. 2023 Oct 13;13:17428. doi: 10.1038/s41598-023-44549-x (PMC10575918; doi:10.1038/s41598-023-44549-x)
Supplement: Supplementary file 2 — Supplementary Information 2. [file 41598_2023_44549_MOESM2_ESM.doc]

STROBE Statement—Checklist of items that should be included in reports of ***case-control studies***

|  | Item No | Recommendation |
| --- | --- | --- |
| **Title and abstract** | 1 | 1. Indicate the study’s design with a commonly used term in the title or the abstract   “A retrospective analysis” has been presented in the title.  In addition, the abstract has also stated: “A total 541 patients who underwent brain tumor resection surgery in our hospital from Jan 2019 to Dec 2021 were respectively enrolled in the study.” |
|  |  | 1. Provide in the abstract an informative and balanced summary of what was done and what was found   Early identification of a patient with a high risk of blood transfusion during brain tumor resection surgery is difficult, but critical for implementing preoperative blood-saving strategies. We aimed to develop and validate a machine-learning prediction tool for intraoperative blood transfusion in brain tumor resection surgery. |
| Introduction | | |
| Background/rationale | 2 | Explain the scientific background and rationale for the investigation being reported  Large amounts of blood loss may occur during these procedures which can frequently result in anemia. Although blood transfusion could increase hemoglobin (HB) levels and improve tissue perfusion, it could result in many transfusion-related complications, such as fever, surgical site infection, acute lung injury; or even prolonged hospital stay and death. In addition, blood shortage is an increasing problem in many countries. Therefore, early identification of high-risk patients is a necessary step for implementing specific interventions preoperatively to save blood resources and improve clinical outcomes. |
| Objectives | 3 | State specific objectives, including any prespecified hypotheses  A prediction tool that could quantitively assess the probability of intraoperative blood transfusion for brain tumor surgery remains to be done. Therefore, our study aimed to develop a machine-learning calculator to predict intraoperative blood transfusion in patients receiving brain tumor surgery and to evaluate its discriminative ability. |
| Methods | | |
| Study design | 4 | Present key elements of study design early in the paper  Patients who received elective brain tumor resection surgery in our tertiary hospital from January 2019 to December 2021 were respectively included for analysis. |
| Setting | 5 | Describe the setting, locations, and relevant dates, including periods of recruitment, exposure, follow-up, and data collection  Locations: xiangyang center hospital; date: from Jan 2019 to Dec 2021. |
| Participants | 6 | 1. Give the eligibility criteria, and the sources and methods of case ascertainment and control selection. Give the rationale for the choice of cases and controls   Inclusion and exclusion criteria have been stated in the second paragraph of the method. Blood transfusion was defined as receiving packed red blood cells intraoperatively. |
|  |  | 1. For matched studies, give matching criteria and the number of controls per case   All the patients who did not received intraoperative blood transfusion were also included for analysis. |
| Variables | 7 | Clearly define all outcomes, exposures, predictors, potential confounders, and effect modifiers. Give diagnostic criteria, if applicable  Patients’ demographic information (gender, age), previous comorbidity (previous cerebral disease, diabetes, cardiovascular disease, pulmonary disease, renal disease, liver disease), brain tumor characteristics (diameter, number of the brain tumor), patient status (ASA, and heart function) and laboratory tests (WBC, HB, PLT, prothrombin time (PT), activated partial prothrombin time (APTT), fibrinogen, D-dimer, total protein (TP), albumin (ALB)) were recorded. These variables were respectively collected from the electronic history database in our hospital and were included for the feature selection during the model development and validation. |
| Data sources/ measurement | 8* | For each variable of interest, give sources of data and details of methods of assessment (measurement). Describe comparability of assessment methods if there is more than one group  The brain tumor was diagnosed by magnetic resonance imaging (MRI). Blood transfusion was defined as receiving packed red blood cells intraoperatively. Intraoperative transfusion was carried out when the hemoglobin level was less than 70 g/L in stable patients and less than 90 g/L in patients with unstable hemodynamics. |
| Bias | 9 | Describe any efforts to address potential sources of bias  These variables were respectively collected from the electronic history database in our hospital and were included for the feature selection during the model development and validation. |
| Study size | 10 | Explain how the study size was arrived at  The sample size selected for benchmarking various machine learning models meets the standard of 10 events per variable |
| Quantitative variables | 11 | Explain how quantitative variables were handled in the analyses. If applicable, describe which groupings were chosen and why  The quantitative data were expressed as mean and standard division (SD) if the data were normally distributed, or else median and interquartile rage. An Independent t-test or Mann-Whitney U test was performed according to the data. |
| Statistical methods | 12 | 1. Describe all statistical methods, including those used to control for confounding   All the statistical methods have been stated in the statistical analysis part. |
|  |  | 1. Describe any methods used to examine subgroups and interactions   We divided the data into development and validation groups. |
|  |  | 1. Explain how missing data were addressed   The missing data were imputed using recursive partitioning and regression trees with 10-fold cross-validation (CV) and then standardized to the same range of values with the max-min method in the training and test sets, respectively. |
|  |  | 1. If applicable, explain how matching of cases and controls was addressed   All the patients who received intracranial brain tumor resection surgery were included. |
|  |  | 1. Describe any sensitivity analyses   We confirmed the efficacy of the nomogram in the validation groups. |
| Results | | |
| Participants | 13* | 1. Report numbers of individuals at each stage of study—eg numbers potentially eligible, examined for eligibility, confirmed eligible, included in the study, completing follow-up, and analysed   Patients’ baseline characteristics were shown in table 1. Finally, a total of 541 patients who received brain tumor resection surgery were included in the analysis, and 141 patients (approximately 26%) received a blood transfusion. |
|  |  | 1. Give reasons for non-participation at each stage   In the study, 6 patients were excluded as they took oral anticoagulants the day before surgery. |
|  |  | 1. Consider use of a flow diagram   See figure 1. |
| Descriptive data | 14* | 1. Give characteristics of study participants (eg demographic, clinical, social) and information on exposures and potential confounders 2. The patients were older in the transfused group compared to the non-transfused group (P < 0.05). Patients with ASA physical status Ⅲ or heart function Ⅱ had a higher incidence of transfusion compared to ASA physical status Ⅰ~Ⅱ or heart function Ⅰ, respectively (P < 0.05). The tumor diameter, PT, and WBC in the transfused group were higher than in the non-transfused group (P < 0.05). Patients’ demographic information (gender, weight), previous co-morbidity (previous cerebrovascular disease, diabetes, cardiovascular disease, pulmonary disease, renal disease, liver disease), tumor characteristics (multi-site brain tumor), and laboratory tests (PLT, activated APTT, fibrinogen, D-dimer, TP, ALB) all were not different between the transfused and non-transfused groups (P > 0.05). |
|  |  | 1. Indicate number of participants with missing data for each variable of interest |
| Outcome data | 15* | Report numbers in each exposure category, or summary measures of exposure  See table 1 |
| Main results | 16 | 1. Give unadjusted estimates and, if applicable, confounder-adjusted estimates and their precision (eg, 95% confidence interval). Make clear which confounders were adjusted for and why they were included   unadjusted estimates were shown in table (the last column is the P for the results). adjusted variables were the seven features and were shown in nomogram. |
|  |  | 1. Report category boundaries when continuous variables were categorized   We did not transform the continuous variables to category variables. |
|  |  | 1. If relevant, consider translating estimates of relative risk into absolute risk for a meaningful time period   We used relative risk to establish the nomogram for predicting blood transfusion. |

| Other analyses | 17 | Report other analyses done—eg analyses of subgroups and interactions, and sensitivity analyses  The prediction tool was established with the seven identified independent relative factors, including HB, diameter, PT, WBC, age, ASA, and heart function |
| --- | --- | --- |
| Discussion | | |
| Key results | 18 | Summarise key results with reference to study objectives  In this study, about one fourths of patients who underwent craniotomy for brain tumor removal received an intraoperative blood transfusion. Seven preoperative indicators, including HB, diameter, PT, WBC, age, ASA classification, and heart function, were identified as relevant factors for intraoperative blood transfusion during brain tumor resection surgery. We established a calculator to predict the intraoperative blood transfusion and the results showed that the prediction model had good discriminative ability. |
| Limitations | 19 | Discuss limitations of the study, taking into account sources of potential bias or imprecision. Discuss both direction and magnitude of any potential bias  However, the present study has some shortcomings: (1) It was adopted to be a retrospective study, some of the variables may affect the transfusion and could not be measured. |
| Interpretation | 20 | Give a cautious overall interpretation of results considering objectives, limitations, multiplicity of analyses, results from similar studies, and other relevant evidence  The prediction tool was established based on our single centre, and the prediction ability of this model needs to be confirmed by external tests in future. |
| Generalisability | 21 | Discuss the generalisability (external validity) of the study results  We established this prediction tool for predicting intraoperative blood transfusion using seven variables, and the prediction tool is simple and easy to use. The common preoperative variables make the prediction tool easy to be generally used in clinics. |
| Other information | | |
| Funding | 22 | Give the source of funding and the role of the funders for the present study and, if applicable, for the original study on which the present article is based  This research is funded by grants from the Natural Science Foundation of Hubei (2023AFD041, 2019CFB411) and Xiangyang, Hubei (No. 2021YL017) and the Doctor Initiation Funding of Xiangyang Central Hospital (2021-Y-03). |

*Give information separately for cases and controls.

**Note:** An Explanation and Elaboration article discusses each checklist item and gives methodological background and published examples of transparent reporting. The STROBE checklist is best used in conjunction with this article (freely available on the Web sites of PLoS Medicine at http://www.plosmedicine.org/, Annals of Internal Medicine at http://www.annals.org/, and Epidemiology at http://www.epidem.com/). Information on the STROBE Initiative is available at http://www.strobe-statement.org.
